# The Effects of Elk Velvet Antler Dietary Supplementation on Physical Growth and Bone Development in Growing Rats

**DOI:** 10.1155/2015/819520

**Published:** 2015-08-23

**Authors:** Jiongran Chen, Yanfei Yang, Sepideh Abbasi, Daryoush Hajinezhad, Saija Kontulainen, Ali Honaramooz

**Affiliations:** ^1^Key Laboratory of New Animal Drug Project, Gansu Province, Key Laboratory of Veterinary Pharmaceutics Discovery, Ministry of Agriculture and Lanzhou Institute of Animal Science and Veterinary Pharmaceutics, Chinese Academy of Agricultural Sciences, Lanzhou 730050, China; ^2^Department of Veterinary Biomedical Sciences, Western College of Veterinary Medicine, University of Saskatchewan, 52 Campus Drive, Saskatoon, SK, Canada S7N 5B4; ^3^College of Kinesiology, University of Saskatchewan, 87 Campus Drive, Saskatoon, SK, Canada S7N 5B2

## Abstract

Elk velvet antler (EVA) has been used in traditional Oriental medicine for centuries to promote general health; however, little evidence for its effect on bone development is available. We investigated the effects of lifelong exposure of Wistar rats to a diet containing 10% EVA on physical growth and bone development. Measurements included weekly body weights, blood chemistry and kidney and testis/ovary indices (sacrificed at 5, 9, or 16 weeks of age), and bone traits of the femur bones by peripheral quantitative computed tomography (pQCT). Mean body weights were higher in the EVA group at 4–8 weeks in males and at 5 weeks of age in females. The kidney indices were greater in EVA dietary supplemented male rats at 5 and 16 weeks of age, in females at 16 weeks of age, and testis/ovary indices at 5 weeks of age. The femoral length was increased in both males and females at 5 weeks, and several pQCT-measured parameters had increased in EVA males and females. The activity of alkaline phosphatase (ALP) increased in EVA group while the content of calcium and phosphorus did not differ among groups. Our results seem to support a role for dietary supplementation of EVA on growth and bone development in this model.

## 1. Introduction

Population-based epidemiological evidence has bolstered a renewed interest in the ancient philosophy of food as medicine, exemplified in Hippocrates's famous dictum “Let food be thy medicine and medicine thy food” [1]. Consequently, a new trend in nutritional research attempts to evaluate empirical information and translate this knowledge into evidence-based molecular nutrition. Although nutritious foods have classically been perceived as a means to provide energy and building material for the body, the notion of food components protecting the body against diseases is becoming better recognized. In particular, research over the past couple of decades has provided evidence for the biological activity of dietary factors that can influence specific molecular systems and pathways that maintain body functions [2, 3].

Velvet antler has been used for various health benefits for centuries and is currently one of the most frequently prescribed animal-derived ingredients in traditional Oriental medicine [1]. Western holistic health and natural product industry has also started promoting antler's use as a food supplement or nonpharmaceutical therapeutic agent for applications in human and veterinary medicine. Farmed North American elk or wapiti (*Cervus canadensis*) or the European red deer (*Cervus elaphus*) are the main sources of antler for commercial use, although other deer species and countries may also be the source of velvet antler for use in traditional Oriental medicine.

Elk velvet antler (EVA) is harvested while the antler is still developing and has a soft, velvety covering [4]. The use of antler is believed to date back to the Han dynasty in China (206 BC to 220 AD). One of the earliest documented historical accounts of using velvet antler has been provided by the 1st century's Roman Plinius Secundus describing antlers as containing “some sort of healing drug” and detailing its use for treatment of epilepsy. Li Shi Zhen's Chinese medical text in the 16th century has also discussed the use of antler in the form of powders, pills, extracts, tinctures, and ointments. In addition to referring to antler as a general tonic and enhancer of sexual virility, Topsell (1607) described the use of powdered antler to benefit such varied conditions as baldness, pimples, toothaches, and even snakebites or lice infestations [5]. Therefore, the traditional beliefs about antler products have persisted for many centuries mostly in the Orient but also in the West. Velvet antler has been suggested to invigorate the kidney's yang, benefit blood and essence, strengthen bones and muscles, modulate Chong and Ren channels, and drain pus [6]. Velvet antler has also been used as an immune modulator and erythropoietic agent to improve blood circulation and muscle strength [7]. Prescribing velvet antler is not limited to adults; it is estimated that about 10% of Korea's velvet antler is used for preventive and restorative purposes in children [8].

In a previous study [9], using a rat model we investigated the effects of long-term maternal dietary supplementation of EVA on development of their offspring. Although the maternal EVA supplementation did not affect the body weight gain of offspring (monitored until 3 weeks of age), it seemed to accelerate certain early life physical, reflexologic, and neuromotor developmental milestones. However, one of the common themes in reference to beneficial effects of EVA is its effect on bone. Velvet antler is thought to possess a bone strengthening activity and has been used in treating bone fractures [10]. Therefore, we hypothesized that dietary supplementation of EVA to young rats would also have beneficial effects on bone development without affecting the body weight. Subsequently, the objective of this study was to investigate the effects of EVA used as dietary supplementation on physical growth and bone development in rats, as a model. Young growing rats serve as a useful model for the study of factors that modulate bone mineral development [11, 12].

## 2. Materials and Methods

### 2.1. Animal Diets

Both diets were fed in powdered form. The control diet was made from regular rodent chow (Prolab RMH 3000, PMI Nutrition International, St. Louis, MO, USA), ground into powder using a laboratory mill. Elk antlers used in this study were harvested in late spring or early summer from live farmed elk raised in Saskatchewan, Canada, while they were still in their velvet stage. Elk velvet antler (EVA) powder (Norelkco Nutraceuticals, Moosomin, SK, Canada) was derived using the freeze-drying technique for antler drying [9]. Briefly, the velvet antler was frozen immediately after harvesting and transported to the plant where about 2 cm of the button end was cut off in order to help control bacteria and the hide was removed. The remaining frozen stick was shredded onto stainless steel trays to expose maximum surface area. The shred was quickly (within 12 h) dried by a blast of cold (−1°C), dry air to minimize loss of bioavailable nutrients and prevent pathogen buildup. The dried shred was then ground in a nitrogen cooled grinder to a fine powder. In our laboratory, a 10% EVA diet was made by adding 1 : 9 EVA powder to powered regular rat chow and mixing to homogeneity. When mixed evenly, the 10% EVA diet appeared slightly reddish-brown compared to the control diet that was yellowish-brown.

### 2.2. Animals and Study Design

Eighteen adult female Wistar rats weighting ~200–250 g and 18 adult male Wistar rats weighting ~400–450 g were obtained (Charles Rivers, Montreal, QC, Canada). Female and male rats were randomly divided into control and treatment groups and housed in individual Plexiglas cages, lined with sawdust in a room with controlled photoperiod (lights on from 06:00 through 18:00), under a constant temperature of 21°C and humidity of 60%. Rats were provided* ad libitum* with water, and regular powdered rat chow (control parents) or 10% EVA-added powdered rat chow (EVA parents) for 2 weeks prior to mating. For breeding, each female rat from each diet group was paired with a stud male rat of the same diet group (i.e., on a 1 : 1 basis) for 5 days or until a vaginal plug was confirmed as evidence of mating. Pregnant female rats were caged individually and checked twice daily until pups were born. The feeding regiments continued for each group through the periods of pregnancy and lactation. All dams were allowed to deliver naturally and rear their young to weaning at 3 weeks of age. While suckling, the pups had access to the diet that their dams were receiving and continuously received the respective diet after weaning; these pups were used in this study. After weaning, sibling pups were kept in groups of 3 or 4 in gender-segregated cages.

In total, 42 male and 42 female rat pups were used for this study. Representative pups from each diet group were sacrificed at different time intervals from early postnatal to young adulthood. This research was approved by the University of Saskatchewan's Animal Research Ethics Board and adhered to the Canadian Council on Animal Care guidelines for humane animal care.

### 2.3. Sample Preparation and Blood Chemistry

Body weights of male and female rats were recorded weekly from birth and through the experimental period. At 5, 9, and 16 weeks of age, 7 male and 7 female rats were randomly selected from each diet group and were anesthetized by intraperitoneal injection of ketamine hydrochloride (75 mg/kg; Ketalene, Bimeda-MTC, Cambridge, ON, Canada) and xylazine hydrochloride (10 mg/kg; Vet-A-Mix, Shenandoah, IA, USA). Rats were sacrificed by exsanguination and blood samples were collected. The blood samples were prepared for the measurement of calcium (Ca), phosphorus (inorganic P), and activity of alkaline phosphatase (ALP) using Roche Hitachi 912 Chemistry Analyzer. The weights of kidneys and testes/ovaries were recorded and the organ index was calculated (% of organ weight/body weight). Left femora were excised, cleaned of all soft tissue, weighted, soaked in saline for 30 sec, wrapped in saline-soaked plastic film bandages, and stored at −80°C in freezer bags. This method of storage has been shown to prevent dehydration and changes to the bone biomechanical properties [13, 14].

### 2.4. Femur Length Measurement

On the day of analysis, the femora were slowly thawed at room temperature while remaining wrapped in the saline-soaked plastic film bandages and were unwrapped only during measurements. A digimatic caliper was used to measure the length of bones. In our laboratory, the root-mean-square coefficient of variation (CVrms) for determination of the femoral length was 0.2%.

### 2.5. Peripheral Quantitative Computed Tomography of Femora

The cross-sections of the femoral diaphysis and distal metaphysis were scanned using peripheral quantitative computed tomography (pQCT, Stratec XCT Research M, software version 5.40B, Stratec Medizintechnik GmbH, Pforzheim, Germany). The selected voxel size was 0.111 mm^3^, scan thickness was 2.4 mm, and the scan speed was 10 mm/s. For the pQCT assessment, the femur was inserted into a custom-made plastic tube with the shaft in axial direction, and one cross-sectional slice was scanned at the 10% (distal metaphysis) and 50% (midshaft diaphysis) sites of the femoral length. At the shaft site, we analyzed cortical bone mineral content (cBMC), cortical bone mineral density (cBMD), and cortical cross-sectional area (cCSA) using the pQCT software with contour mode 1 (threshold 480 mg/cm^3^). In our laboratory, the CVrms in the femoral midshaft were 1.5% for the cCSA, and 0.6% for the cBMD.

The scan at the distal metaphysis (at 10% site of the femoral length) was used to determine the total cross-sectional area (tCSA), total bone mineral content (tBMC), and total bone mineral density (tBMD) using contour mode 1 (threshold 169 mg/cm^3^). In our laboratory, the CVrms were 3.9% for tCSA, 2.2% for tBMC, and 2.1% for tBMD.

### 2.6. Femur Ash Preparation and Measurement

After scanning, the femur samples were dried to a constant weight at 105°C and then ashed in a muffle furnace at 600°C for 12 h [15]. The percent ash was calculated by dividing the ash weight by the weight of the specimen in air.

### 2.7. Statistical Analyses

Statistical analysis was performed using SPSS for Windows (SPSS 17.0; SPSS, Chicago, IL, USA). A two-way analysis of variance (ANOVA) statistical test was performed for the effects of diet and age. For the body weight data, differences were assessed using repeated measures ANOVA. For the organ index data, percentages were transformed (using Arcsin function) prior to analysis using ANOVA. All other comparisons were made using the Student* t*-test. A minimum *P* value of 0.05 was considered statistically significant.

## 3. Results

### 3.1. Food Consumption and General Observations

Both the control powdered chow and the diet supplemented with 10% EVA were readily consumed by the male and female rats. We did not notice any obvious differences in the weight of food consumed by the rats of comparable groups. We also did not detect any visible changes in behavior or toxic/teratogenic signs in parents or offspring rats consuming the EVA supplemented diet and no postnatal deaths occurred during the experimental period.

### 3.2. Body Weight and Organ Indices

**Figure 1:**
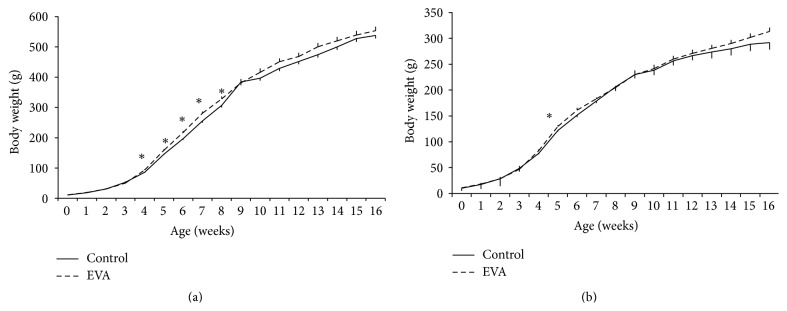
Mean body weights of rats receiving either a control or EVA supplemented diet. Mean (± SEM) body weights of male (a) and female (b) rats, measured weekly from birth until 16 weeks of age (end of study). Groups of 7 male and 7 female rats from each of the control and EVA groups were sacrificed at 5, 9, or 16 weeks of age; therefore, *n* = 21/group until 5 weeks, *n* = 14/group until 9 weeks, and *n* = 7/group until 16 weeks of age. Asterisks indicate significant differences as compared to age-matched controls (^*∗*^ *P* < 0.05).

Growth curves of male and female rats in both groups are shown from birth to 16 weeks of age (end of experiment) in Figure 1. The general trend in body weight increases was the same for animals of each gender for control and EVA groups. Male rats in both groups were heavier than female rats of comparable age. As compared to age-matched control males, EVA supplemented males were heavier by an average of 8.7% at 4 weeks, 9.3% at 5 weeks, 10.5% at 6 weeks, 9.9% at 7 weeks, and 7.0% at 8 weeks of age (*P* < 0.05, Figure 1(a)). In female rats, the body weights in EVA group were higher by an average of 6.3% at 5 weeks of age than age-matched controls (*P* < 0.05, Figure 1(b)).

Table 1 contains a summary of organ (kidney and testis/ovary) index data for different dietary and age groups. The kidney indices were greater in EVA supplemented male rats at 5 and 16 weeks of age and in females at 5 weeks of age than age-matched controls (*P* < 0.05). The testis/ovary indices of EVA supplemented group of male/female rats were also greater at 5 weeks of age than age-matched control groups (*P* < 0.05, Table 1).

### 3.3. Geometric and Densitometric Parameters of the Femur Bone

The geometric and densitometric parameters for femora of rats in both groups at different ages are summarized in Table 2. The femur length increased in both control and EVA groups of male and female rats over the observation period (*P* < 0.05, measured at 5, 9, or 16 weeks of age). Male rats in both control and EVA groups showed a greater increase in femur length over time as compared to age-matched females (*P* < 0.05). Femora were longer in 5-week-old males and females of EVA group than their respective age-matched controls (*P* < 0.05, Table 2).

All geometric (cross section area) and densitometric bone parameters (bone mineral content and density) measured at the femoral distal metaphysis and midshaft diaphysis increased in both male and female rats over time (*P* < 0.05). Several of these parameters were higher in the EVA supplemented groups than controls. In males, at 5 weeks of age, the tCSA; at 9 weeks of age, the tBMD; and at 16 weeks of age, the tBMC, tCSA, cBMC, and tCSA were greater in EVA rats than age-matched controls (*P* < 0.05). In females, at 5 weeks of age, the tBMC, tBMD, tCSA, cBMC, cBMD, and cCSA of the femur; at 9 weeks of age, the tBMC and tBMD; and at 16 weeks of age, the tBMD were greater in EVA rats than in age-matched controls (*P* < 0.05, Table 2).

Percentages of femur bone ashes were higher in the EVA groups of female rats at 5 and 16 weeks of age than in age-matched controls (*P* < 0.05), but in males, these percentages did not differ between the dietary groups (*P* > 0.05, Table 2).

### 3.4. Calcium, Phosphorus, and Alkaline Phosphatase

The serum content of calcium and phosphorus and serum activity of alkaline phosphatase (ALP) are presented in Table 3. Serum content of calcium and phosphorus did not differ between EVA and age-matched control groups in either male or female rats (*P* > 0.05). The activity of ALP in serum was higher in both male and female EVA rats at 5 and 16 weeks of age than in age-matched controls (*P* < 0.05, Table 3).

## 4. Discussion

Despite the historic importance and continued widespread use of velvet antler in traditional Oriental medicine, few studies have examined the virtue of its potential effects. Assessment of EVA effects on physical growth and bone development is important and relevant because EVA contains a number of bioactive substances that are believed to possess bone-promoting properties [10]. Therefore, this study was designed to evaluate the effects of long-term dietary supplementation of EVA on physical growth and bone development of rats as a model. To the best of our knowledge, this is the first controlled study investigating the long-term (from before conception to adulthood) effects of EVA dietary supplementation on bone development in intact rats. We assessed physical growth parameters (including body growth curves and organ indices), bone development parameters (including measurements of femur bone geometric and densitometric properties, dry matter content ash), and bone related serum mineral and enzyme levels (calcium, phosphorus, and alkaline phosphatase). In all comparisons, we also assessed the potential gender differences because a number of studies have highlighted differences related to gender in development of progeny from dams undergoing experimental dietary or drug interventions [16–21].

The dose and route of EVA supplementation were based on our previous study in which dietary EVA supplementation of rat dams improved the acquisition of neurological reflexes and accelerated the physical and neuromotor development of rat offspring, without causing discernible adverse effects [9]. In the same report, we observed that the rate of body weight gain monitored until 3 weeks of age did not differ between pups nursing mothers receiving regular diet and those receiving EVA supplemented diet [9]. In the present study, however, we continued to measure body weights from birth to postpuberty. Our results showed that, within gender, rats receiving dietary EVA supplementation had an overall growth curve pattern that was similar to controls but EVA rats gained higher body weights at certain time periods during development (i.e., 4–8 weeks of age in males and 5 weeks of age in females).

The organ indices (relative weights) of kidneys and testes/ovaries were also higher at two of the three examination points (i.e., at 5 and 16 weeks of age) in EVA groups of rats. During normal growth and development, many physical and physiological factors influence bone mass accumulation, some of the most important of which include body weight, sex hormone status, nutrition, and exercise [22, 23]. Our observation of the effect of EVA on weight gain in young Wistar rats is different from a previous report in which providing a diet containing 10% EVA starting from late gestation to 12 weeks of age did not affect body weight of Fisher rats [12]. The reason for the discrepancy between our results and the latter study is not clear but it could relate to the use of different breeds of rats or that we had supplemented maternal EVA for a longer period of time/development (at least 2 weeks prior to mating).

Maturation of the testis/ovary, by releasing sex hormones, also positively influences bone mass accumulation. In the present study, we observed increased testis/ovary indices in EVA groups of male and female rats only at 5 weeks of age. In Wistar rats, the first significant increases in testosterone and estradiol are expected to occur at about 5 weeks of age or shortly after [24, 25]. This observation may indicate that EVA dietary supplemented rats had achieved an earlier gonadal maturation (as evident by increased gonadal indices) as compared to age-matched controls. Interestingly, the improved body growth of male and female rats of the EVA groups also occurred around the time of sexual maturation, while for the later time points their body weights were not different from controls. The earlier increase in gonadal weight indices may have also contributed to the improved bone parameter in the EVA groups.

In the present study, we also examined the geometric and densitometric parameters of the femora in male and female rats as an indication of their bone development. Epidemiologically, adverse growth conditions early in life are related to lower bone mass later in childhood [26] and in old age [27]. A systematic review confirmed that birth weight is positively associated with adult bone mass [28]. At birth, the skeletal size of infants categorized as small size for gestational age (SGA) is lower than appropriately sized infants, likely as a result of abnormal development of the epiphyseal growth plate [29, 30] or reduced femoral cortical thickness (25%) and diaphysis diameter (25%) [13].

One of the most basic densitometric parameters is bone mineral content (BMC) which is also one of the main determinants of the mechanical strength of bone [16, 31]. BMC is defined as either the mass of minerals contained in the entire bone (g) or as the mass of minerals per unit bone length (g/cm or mg/mm). Although mineral mass can be expected to be a suitable proxy for bone stability, BMC is obviously a size-dependent parameter. This is a drawback, because short children will have a lower BMC than their healthy age-matched peers, even if their (smaller) bones are otherwise completely normal. The bone mineral density (BMD) is used to evaluate the bone mineral content (BMC) for a given bone volume and its measurement is important for assessing bone metabolism. Therefore, bone densitometry techniques are increasingly added to other methodologies (e.g., specific blood or urine biochemical markers of bone resorption) to investigate bone metabolism in animal models [17–21, 32].

The same reasoning applies to the areal bone mineral density (BMD), which is also a widely used densitometric parameter [33, 34]. Areal BMD is defined as the mineral mass of a bone divided by its projection area in a given direction (g/cm^2^) and is directly related to the mean path length that the radiation beam takes through the bone [33, 35, 36]. Therefore, similar to BMC, areal BMD is often difficult to interpret in children and adolescents with short stature.

In the present study, the femora in 5-week-old EVA supplemented rats were longer than those of control rats. Several geometric and densitometric parameters of the femur bone were also higher in EVA groups of rats than age-matched controls at different ages. These effects were especially highlighted in 5-week-old female and 16-week-old male groups of EVA supplemented rats. This may suggest that female pups benefited in several parameters (bone content, density, and cross-sectional area) from the dietary EVA, which may have promoted bone growth earlier in females than in males. This may be related to differences in sexual hormones, indicated by higher gonadal indices but not directly measured in this study.

Low BMD is the single best predictor of fracture risk [37, 38]. In fact, BMD of the spine remained 1.3 standard deviations below normal in 9-year-old children who were born SGA [39]. Young adult males born SGA exhibited a bone mineral turnover rate that was twofold that of males born with a normal weight [40], while for the elderly, bone mineral content of the proximal femur (hip) and lumbar spine was directly correlated with birth weight [41, 42]. In our study, dietary supplementation of EVA from at least 2 weeks prior to conception through maturity had an obvious effect on the femur growth suggesting that an early life dietary supplementation of EVA may be beneficial in achieving the trajectory for bone mass following limited intrauterine growth. These results, however, would require further investigation as to the safety and feasibility of EVA use in primate models before any recommendation for human consumption can be made.

Changes in the serum concentrations of calcium and phosphorus directly influence the calcification and dissolving of bone and can be measured as parameters associated with bone metabolism. Therefore, we also examined the impact of EVA dietary supplementation on the enzyme ALP and the content of Ca and P in serum. ALP is a membrane-bound enzyme found in abundance in the bone, liver, kidney, intestine, and placenta. ALP is also one of the earlier markers in the maturation of osteoblasts [43–46]. Osteoblasts can secrete ALP, which permeates into blood and therefore its serum concentration rise. Previous studies showed that the levels of ALP activity in osteoblast cell lines and bones could be correlated with the rate of collagen production and with the rate of ALP activity release from the cells into the culture medium. It has also been reported that the amount of skeletal ALP activity in serum could be correlated with the rate of bone formation [47–50]. Serum concentration of calcium and phosphorus in balance can be responsible for bone growth [51]. In our study, significant increases of ALP in serum were observed in rat receiving EVA dietary supplementation at 5 and 16 weeks of age, while the content of Ca and P in serum did not change compared to age-matched controls. These ages (5 and 16 weeks) are incidentally the same ages when most of the changes in geometric and densitometric bone parameters also occurred. Therefore, the observed higher concentrations of ALP in serum (which may imply an increased osteoblast activity), along with the balanced contents of serum calcium and phosphorus, indicate that the supplemented EVA in diet may have played an important role in stimulating ALP activity to promote the action of osteoblasts and retain the balance for calcium and phosphorus in the blood.

It is also possible that one or several components in EVA dietary supplementation have initiated a direct modulating effect on the bone growth. EVA has been reported to contain all essential amino acids, collagen, hyaluronic acid, glycosaminoglycan and chondroitin sulfate, polysaccharides, and a number of fatty acids, notably C18:3-omega-6 fatty acid. EVA is also high in calcium, phosphorus, iron, and zinc [12]. In EVA, amino acids such as glycine, alanine, proline, and glutamic acid are found proportionally higher than histidine, isoleucine, leucine, serine, tyrosine, and lysine. Classes of lipid found in antlers include fatty acids, glycolipids, and phospholipids. Lysophosphatidylcholine with palmitic acid (C16:0) accounts for 50% of total fatty acids isolated from velvet antlers [52]. It is reported that the composition of lipids such as prostaglandin, phospholipids, and polyunsaturated fatty acids changed at different stages of antler growth [53]. Other than organic compounds, velvet antlers contain minerals such as Ca, P, Mg, Fe, K, and Na as major components and Ni, Cu, Ti, Mn, Sn, Pb, Si, and Ba as minor components.

Elk velvet antlers have been shown to contain chondroitin sulfate as a major glycosaminoglycan with small amounts of keratan sulfate, hyaluronic acid, and dermatan sulfate [54, 55]. Glycosaminoglycans found in water-soluble fractions of velvet antlers showed a growth promoting effect on cells [56, 57]. Cartilaginous portion of EVA also contains proteoglycans chondroitin sulfate (as 90% of total proteoglycans in EVA) and decorin [58]. It is believed that cartilage proteoglycans regulate water retention and differentiation and proliferation of chondrocytes in the cartilage tissue. Four types (I, II, III, and X) of collagens have also been identified and immunohistologically localized in antlers [59].

Feng et al. identified a number of growth factor families in antler extract, including bone morphogenetic proteins (BMPs) [60, 61] and fibroblast growth factors (FGFs) [10]. Members of these families have powerful anabolic effects on bone. BMPs have powerful local effects on bone formation [62, 63]. FGFs systemically stimulate bone formation, restore trabecular bone microarchitecture, and enhance fracture repair [64, 65].

Kim et al. reported that the intertrabecular connections were well maintained and bone loss was absent upon administration of osteoporosis-induced rats with antler extract [66]. In addition, it was reported that deer antler aqua-acupuncture (DAA) had antibone resorption activity in adjuvant-induced arthritic rats [67].

In the present study, we evaluated the effect of EVA dietary supplementation in both male and female rats. Although we observed similar patterns for the majority of measured outcomes between male and females rats, there were instances where gender differences were noted (such as age in which maximal changes in bone parameters occurred). There is a considerable new body of data suggesting that important differences exist in the bioavailability, metabolism, distribution, and elimination of foods and beverages in males and females [2]. Obvious differences exist between the sexes in the structural components of bone strength (e.g., skeletal dimensions and cortical thickness), biomechanical responses, mineral mass, and turnover [2], yet the differential impact of diet supplementation on males and females has received relatively little attention.

As a whole, the observed positive effect of EVA on bone development in the present study is consistent with some of the account originated from the principles of traditional Chinese medicine about velvet antler. The exact mechanisms of action and biologically active substances responsible for such effects would require further research to be elucidated.

## 5. Conclusions

In summary, our study of the effects of EVA dietary supplementation on body weight and femur development in growing rats tends to be supportive of the beneficial effects of EVA on bone development. Considering the traditional use of EVA as a tonic and valuable drug in Oriental medicine with long history, these findings provide support for continued exploration of nutraceuticals or functional foods.

## Figures and Tables

**Table 1:** Organ indices (percentage of organ weight/body weight) of male and female rats receiving either a control or elk velvet antler (EVA) supplemented diet.

	Age					
	5 weeks		9 weeks		16 weeks	
	Group					
	Control	EVA	Control	EVA	Control	EVA
	Male					
Kidney index (%)	9.36 ± 0.11	10.71 ± 0.22^*∗∗*^	7.39 ± 0.26	7.06 ± 0.30	6.56 ± 0.25	7.47 ± 0.26^*∗*^
Testis index (%)	7.42 ± 0.13	8.32 ± 0.19^*∗∗*^	8.09 ± 0.18	7.69 ± 0.31	6.73 ± 0.23	7.28 ± 0.27

	Female					
Kidney index (%)	9.05 ± 0.14	9.67 ± 0.66	7.85 ± 0.40	8.19 ± 0.08	6.48 ± 0.13	8.20 ± 0.34^*∗∗∗*^
Ovary index (%)	0.59 ± 0.03	0.74 ± 0.03^*∗∗*^	0.59 ± 0.04	0.59 ± 0.03	0.59 ± 0.04	0.55 ± 0.02

Data are presented as mean ± SEM (*n* = 7/group/age).

Asterisks indicate significant differences as compared to age-matched controls (^*∗*^ *P* < 0.05; ^*∗∗*^ *P* < 0.01; ^*∗∗∗*^ *P* < 0.001).

**Table 2:** Geometric and densitometric femur bone measurements at different ages in male and female rats receiving either a control or elk velvet antler (EVA) supplemented diet.

	Age					
	5 weeks		9 weeks		16 weeks	
	Group					
	Control	EVA	Control	EVA	Control	EVA
	Male					
Femur length (mm)	25.07 ± 0.33	26.07 ± 0.38^*∗*^	35.07 ± 0.38	35.5 ± 0.32	39.75 ± 0.37	40.14 ± 0.26
Femur metaphysis						
tBMC (mg/mm)	6.80 ± 0.24	7.36 ± 0.40	19.22 ± 0.70	20.98 ± 1.04	28.06 ± 1.08	31.79 ± 0.28^*∗*^
tBMD (mg/cm^3^)	276.12 ± 8.24	277.25 ± 11.69	455.14 ± 7.29	489.21 ± 20.00^*∗*^	610.03 ± 16.55	610.10 ± 19.92
tCSA (mm^2^)	24.64 ± 0.65	26.49 ± 0.45^*∗*^	42.20 ± 1.20	42.90 ± 1.15	46.08 ± 1.63	52.44 ± 1.70^*∗*^
Femur diaphysis						
cBMC (mg/mm)	2.27 ± 0.18	2.42 ± 0.20	7.70 ± 0.25	8.05 ± 0.26	11.98 ± 0.29	12.95 ± 0.20^*∗*^
cBMD (mg/cm^3^)	760.47 ± 16.05	781.44 ± 12.12	1080.17 ± 7.78	1086.70 ± 8.20	1219.41 ± 8.00	1222.97 ± 7.62
cCSA (mm^2^)	2.96 ± 0.19	3.08 ± 0.23	7.13 ± 0.22	7.41 ± 0.21	9.83 ± 0.26	10.59 ± 0.18^*∗*^
Bone ash (% of DM)	59.51 ± 0.71	59.86 ± 0.90	64.91 ± 0.53	65.80 ± 0.51	68.45 ± 0.53	67.68 ± 0.48

	Female					
Femur length (mm)	24.50 ± 0.22	25.00 ± 0.00^*∗*^	32.25 ± 0.40	32.75 ± 0.15	35.00 ± 0.42	35.92 ± 0.44
Femur metaphysis						
tBMC (mg/mm)	6.21 ± 0.48	7.99 ± 0.35^*∗*^	18.25 ± 0.54	19.75 ± 0.26^*∗*^	25.07 ± 0.47	25.81 ± 1.06
tBMD (mg/cm^3^)	273.24 ± 10.12	304.29 ± 7.14^*∗*^	519.58 ± 4.93	557.60 ± 6.71^*∗∗*^	670.74 ± 9.65	709.10 ± 10.20^*∗∗*^
tCSA (mm^2^)	22.55 ± 1.03	25.54 ± 0.58^*∗*^	35.15 ± 1.14	35.45 ± 0.55	37.41 ± 0.70	36.33 ± 1.09
Femur diaphysis						
cBMC (mg/mm)	2.18 ± 0.07	2.71 ± 0.07^*∗∗∗*^	6.29 ± 0.22	6.52 ± 0.11	8.51 ± 0.17	8.88 ± 0.35
cBMD (mg/cm^3^)	775.31 ± 18.28	832.04 ± 9.20^*∗*^	1089.20 ± 11.14	1084.11 ± 8.90	1214.47 ± 6.82	1220.32 ± 12.75
cCSA (mm^2^)	2.82 ± 0.08	3.26 ± 0.07^*∗∗∗*^	5.78 ± 0.22	6.01 ± 0.10	7.00 ± 0.11	7.26 ± 0.23
Bone ash (% of DM)	59.63 ± 1.40	63.42 ± 0.89^*∗*^	66.66 ± 0.48	66.94 ± 0.19	67.64 ± 0.58	69.43 ± 0.07^*∗∗*^

Data are presented as mean ± SEM (*n* = 7/group/age).

Asterisks indicate significant differences as compared to age-matched controls (^*∗*^ *P* < 0.05; ^*∗∗*^ *P* < 0.01; ^*∗∗∗*^ *P* < 0.001).

tBMC, total bone mineral content; tBMD, total bone mineral density; tCSA, total cross-sectional area; cBMC, cortical bone mineral content; cBMD, cortical bone mineral density; cCSA, cortical cross-sectional area; DM, dry matter.

**Table 3:** Serum content of calcium and phosphorus and activity of alkaline phosphatase at different ages in male and female rats receiving either a control or elk velvet antler (EVA) supplemented diet.

	Age					
	5 weeks		9 weeks		16 weeks	
	Group					
	Control	EVA	Control	EVA	Control	EVA
	Male					
Ca (mmol/L)	2.81 ± 0.03	2.75 ± 0.05	3.09 ± 0.15	2.95 ± 0.06	2.63 ± 0.04	2.48 ± 0.15
P (mmol/L)	3.26 ± 0.10	3.59 ± 0.18	2.96 ± 0.23	2.93 ± 0.38	2.37 ± 0.13	2.34 ± 0.06
ALP (u/L)	226.43 ± 14.44	429.14 ± 38.77^*∗∗∗*^	259.71 ± 12.15	253.71 ± 16.60	111.20 ± 3.10	143.83 ± 8.46^*∗∗*^

	Female					
Ca (mmol/L)	2.73 ± 0.02	2.78 ± 0.04	3.10 ± 0.07	3.04 ± 0.06	2.70 ± 0.03	2.72 ± 0.09
P (mmol/L)	2.92 ± 0.18	3.22 ± 0.06	2.78 ± 0.02	2.94 ± 0.12	2.40 ± 0.18	2.39 ± 0.11
ALP (u/L)	189.14 ± 5.86	350.29 ± 35.37^*∗∗∗*^	159.71 ± 30.87	181.71 ± 26.16	80.43 ± 7.13	115.57 ± 6.45^*∗∗*^

Data are presented as means ± SEM (*n* = 7/group/age). ^*∗∗*^ *P* < 0.01; ^*∗∗∗*^ *P* < 0.001, versus age-matched control group. Ca, calcium; P, phosphorus; ALP, alkaline phosphatase.

## Abbreviations

EVA: Elk velvet antler
Ca: Calcium
P: Phosphorus
ALP: Alkaline phosphatase
pQCT: Peripheral quantitative computed tomography
cBMC: Cortical bone mineral content
cBMD: Cortical bone mineral density
cCSA: Cortical cross-sectional area
tCSA: Total cross-sectional area
tBMC: Total bone mineral content
tBMD: Total bone mineral density.
